# A prenatal interruption of DISC1 function in the brain exhibits a lasting impact on adult behaviors, brain metabolism, and interneuron development

**DOI:** 10.18632/oncotarget.21381

**Published:** 2017-09-28

**Authors:** Dazhi Deng, Chongdong Jian, Ling Lei, Yijing Zhou, Colleen McSweeney, Fengping Dong, Yilun Shen, Donghua Zou, Yonggang Wang, Yuan Wu, Limin Zhang, Yingwei Mao

**Affiliations:** ^1^ Department of Emergency, The People’s Hospital of Guangxi Zhuang Autonomous Region, Nanning, Guangxi, China; ^2^ Department of Biology, Pennsylvania State University, University Park, PA, USA; ^3^ Department of Neurology, First Affiliated Hospital, Guangxi Medical University, Nanning, Guangxi, China; ^4^ Health Examination Center, The People’s Hospital of Guangxi Zhuang Autonomous Region, Nanning, Guangxi, China; ^5^ Department of Neurology, The First People’s Hospital of Nanning, Nanning, Guangxi, China; ^6^ Department of Neurology, School of Medicine, Renji Hospital, Shanghai Jiaotong University, Shanghai, China; ^7^ CAS Key Laboratory of Magnetic Resonance in Biological Systems, State Key Laboratory of Magnetic Resonance and Atomic and Molecular Physics, National Centre for Magnetic Resonance in Wuhan, Wuhan Institute of Physics and Mathematics, Chinese Academy of Sciences, Wuhan, China

**Keywords:** DISC1, interneuron, neural progenitors, metabolism, depression, Pathology Section

## Abstract

Mental illnesses like schizophrenia (SCZ) and major depression disorder (MDD) are devastating brain disorders. The SCZ risk gene, disrupted in schizophrenia 1 (*DISC1*), has been associated with neuropsychiatric conditions. However, little is known regarding the long-lasting impacts on brain metabolism and behavioral outcomes from genetic insults on fetal NPCs during early life. We have established a new mouse model that specifically interrupts *DISC1* functions in NPCs *in vivo* by a dominant-negative DISC1 (DN-DISC1) with a precise temporal and spatial regulation. Interestingly, prenatal interruption of mouse *Disc1* function in NPCs leads to abnormal depression-like deficit in adult mice. Here we took a novel unbiased metabonomics approach to identify brain-specific metabolites that are significantly changed in DN-DISC1 mice. Surprisingly, the inhibitory neurotransmitter, GABA, is augmented. Consistently, parvalbumin (PV) interneurons are increased in the cingulate cortex, retrosplenial granular cortex, and motor cortex. Interestingly, somatostatin (SST) positive and neuropeptide Y (NPY) interneurons are decreased in some brain regions, suggesting that DN-DISC1 expression affects the localization of interneuron subtypes. To further explore the cellular mechanisms that cause this change, DN-DISC1 suppresses proliferation and promotes the cell cycle exit of progenitors in the medial ganglionic eminence (MGE), whereas it stimulates ectopic proliferation of neighboring cells through cell non-autonomous effect. Mechanistically, it modulates GSK3 activity and interrupts Dlx2 activity in the Wnt activation. In sum, our results provide evidence that specific genetic insults on NSCs at a short period of time could lead to prolonged changes of brain metabolism and development, eventually behavioral defects.

## INTRODUCTION

Mental disorders, including SCZ, are chronic and debilitating conditions that have a high prevalence in the population worldwide [1] and no effective treatments available. The lifetime risk of suicide in patients with psychiatric disorder is high [2]. They are among the top ten leading causes of disability (WHO).

The neurodevelopmental theory proposes that a brain defect is inherited or sustained early in life, but is not fully expressed until adolescence [3-5]. Evidences from longitudinal *in-vivo* imaging studies on high-risk subjects have revealed that progressive structural changes in brain precede the onset of symptoms [6-10], and moreover, that these changes continue to progress after the onset of psychosis. Epidemiological studies have revealed that the prenatal period is vulnerable to mental disorders [11-19].

Both genetic and environmental factors are believed to contribute to the risk of psychiatric disorders. Genetic disruptions during the prenatal stage may influence early brain development, including NPC proliferation, differentiation, migration and synaptic formation, and render susceptibility to mental disorders [20]. Among the genetic factors associated with schizophrenia, the *DISC1* gene is disrupted by a balanced chromosomal translocation (1;11)(q42;q14.3) in a Scottish pedigree with a high incidence of major depression, schizophrenia and bipolar disorder [21]. The association of *DISC1* gene with major mental illness [22-26] has been confirmed and replicated in numerous independent genetic studies [26-31]. Although to date, there is a lack of convincing evidence for common variation identified from genome-wide association studies, the high penetrance of the translocation in the original Scottish family [21, 32] and a frameshift mutation in an American family [33], supports that large rare structural mutations in *DISC1* gene may be a significant risk factor. Consistent with this notion, recently *DISC1* deletion has been linked to agenesis of the corpus callosum [34]. Mouse models for *DISC1* have been generated using different promoters, and a variety of phenotypes have been observed. Mice expressing either a transgene of human *DISC1* (mimicking the Scottish translocation mutant) or point mutations by ENU mutagenesis, exhibit increased ventricle size, decreased gray matter volume, changes in dendritic morphology in neurons, and reduced neurogenesis [35-39]. These mice also exhibit behavioral abnormalities such as hyperactivity [35, 36], increased immobility in the forced swim test [35], decreased sociability [36], and decreased working memory [36, 39].

Our previous work identified DISC1 as a key regulator of NPC proliferation and mouse behavior through modulating the canonical Wnt signaling pathway [40]. DISC1 regulates cortical NPC proliferation and neuronal differentiation *via* inhibition of GSK3β. Moreover, human variants of *DISC1* disrupt Wnt signaling during development [41]. Previous studies have generally focused the neuronal disruption of mental illnesses and built animal models based on genetic modifications of neurons. However, many available *DISC1* mouse models use either constitutive neuronal promoters [35, 36, 42-45] or endogenous *Disc1* promoters [37, 46, 47] lacking spatial and temporal control of transgene expression. To overcome this limitation, we established a new Nes-DN-DISC1 transgenic mouse model, which will allow us to monitor the effect of risk genes on NPCs at the beginning of brain development and the long-term effect on neurons. This new mouse model allows us to control the timing and length of DN-DISC1 expression with a spatial distribution specific to NPCs.

Metabonomics profiling has been used for detecting the metabolic information associated with progression of many diseases, such as cancer and diabetes. Rather than transcriptomic profiling, data analysis of spectroscopic data generated from nuclear magnetic resonance (NMR) captures changes of small-molecule metabolite in animal models of mental disorders and offers the potential to characterize specific metabolic phenotypes associated with disrupted behaviors. Yet, no studies have directly investigated the effect that disruption of *DISC1* function may have on the metabolic profile. Our mouse model demonstrated that a short-term interruption of embryonic NPC function by DN-DISC1 exhibited a long term impact on behavioral changes and brain metabolism in adult. Thus, our research provides a different strategy to probe the pathophysiology of mental illness, which will deepen our understanding of the developmental origins of mental diseases.

## RESULTS

### Establishment of a new Nes-DN-DISC1 transgenic mouse model

**Figure 1:**
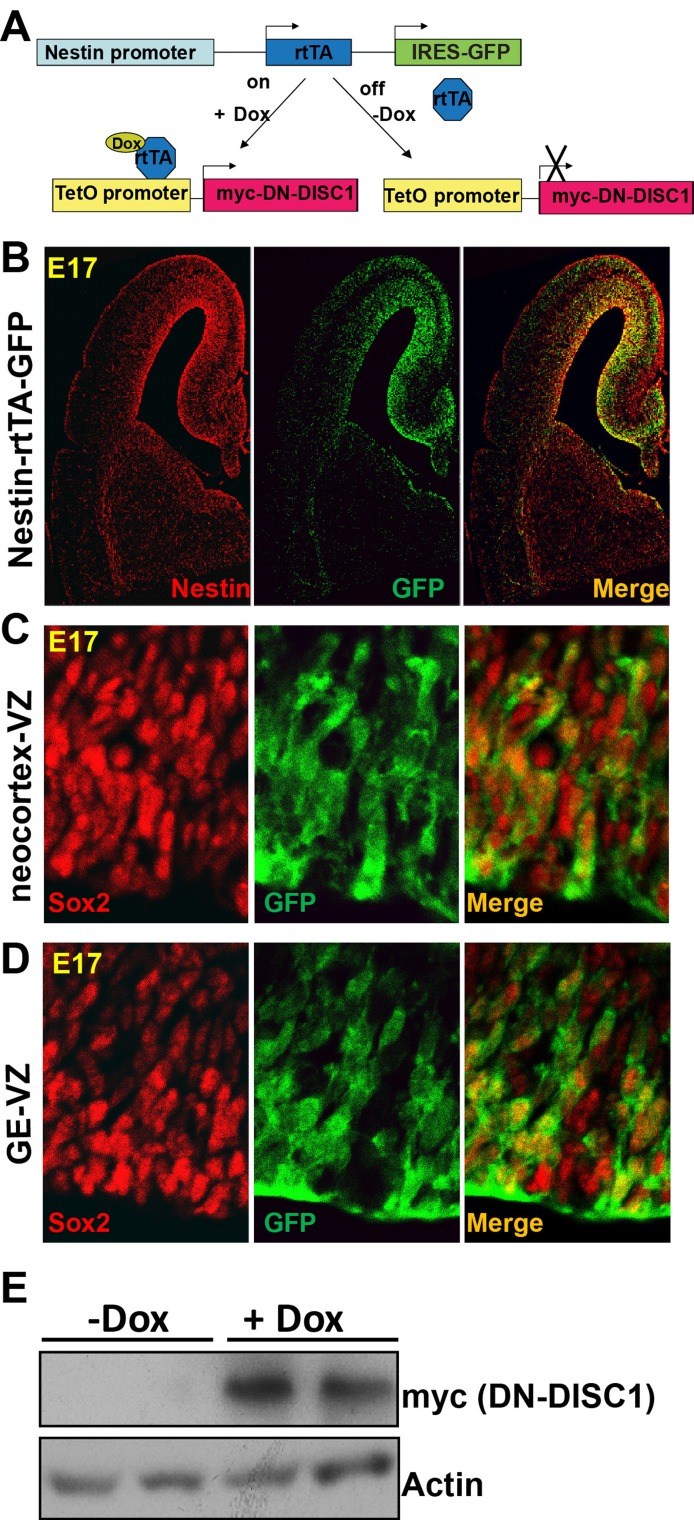
A novel model for dissecting the neural and developmental basis of mental illnesses **A.** Generation of Nes-DN-DISC1 double transgenic mouse line. GFP and rtTA are driven by the nestin promoter, which provides spatial control and only turns on in NPCs. Dox provides a temporal control of DN-DISC1(myc tagged) expression. **B.** Immunostaining of Nes-rtTA-GFP mouse brain with nestin (red) and GFP (green) at E17. **C.** Immunostaining of Nes-rtTA-GFP mouse brain with Sox2 (red) and GFP at the VZ of the neocortex. **D.** Immunostaining of Nes-rtTA-GFP mouse brain with Sox2 (red) and GFP at the VZ of the GE. **E.** Western blot of Nes-DN-DISC1 mice shows that DN-DISC1 (myc tag) is induced at E17. Notably, the basal level of DN-DISC1 is low.

Since *DISC1* has been identified as a genetic risk for multiple mental disorders, several animal models based on DISC1 have been established using either constitutive neuronal promoters [35, 36, 42-45] or endogenous *DISC1* promoters [37, 46, 47]. To implement the spatial and temporal control of transgene expression, we established a new Nes-DN-DISC1 transgenic mouse model by crossing Nes-rtTA transgenic mice [48], in which GFP and rtTA are driven by the nestin promoter, with tetO-DN-DISC1 *mouse* line [36], in which DN-DISC1 is controlled by the doxycycline (Dox) inducible promoter (tetO) (Figure 1A). This mouse line provides a spatial control because the transgene is only turned on in NPCs by the nestin promoter. Dox provides a temporal control of DN-DISC1 expression.

The Nes-rtTA mouse line provides a GFP reporter to specifically label the NPCs in the embryonic brain. The neocortical NPCs of the ventricular zones (VZ) and subventricular zones (SVZ) generate cortical projection neurons. The cortical interneurons from interneuron progenitor cells (IPCs) in the ganglionic eminence (GE) migrate tangentially across areal boundaries of developing cortex, where they mature to form a functional network with excitatory neurons [49]. First, to confirm that GFP labels the same cells as endogenous nestin, we stained the brain of Nes-rtTA mice at embryonic day 17 (E17) with nestin and verified that GFP and nestin showed overlapping expression (Figure 1B). We found GFP-labeled cells in the VZ of the hippocampus, neocortex, and GE, indicating that rtTA is expressed in the NPCs in these three regions. Second, to examine the extent to which GFP+ cells are NPCs, we co-stained brain sections with the NPC marker-Sox2 and GFP and confirmed that all GFP-positive cells in the VZ of the neocortex and GE are Sox2+ NPCs (Figure 1C and 1D). Third, to further confirm DN-DISC1 expression on NPCs, we induced DN-DISC1 expression at the beginning of pregnancy by feeding the mother Dox-containing food. Dox can pass through the placenta [50] and successfully induce DN-DISC1 expression in the embryonic brain of Nes-DN-DISC1 mice (Figure 1E). In contrast, the mice received regular food without Dox did not express detectable DN-DISC1.

### Effects on behavioral changes after a prenatal disruption of DISC1 function in NPCs

**Figure 2:**
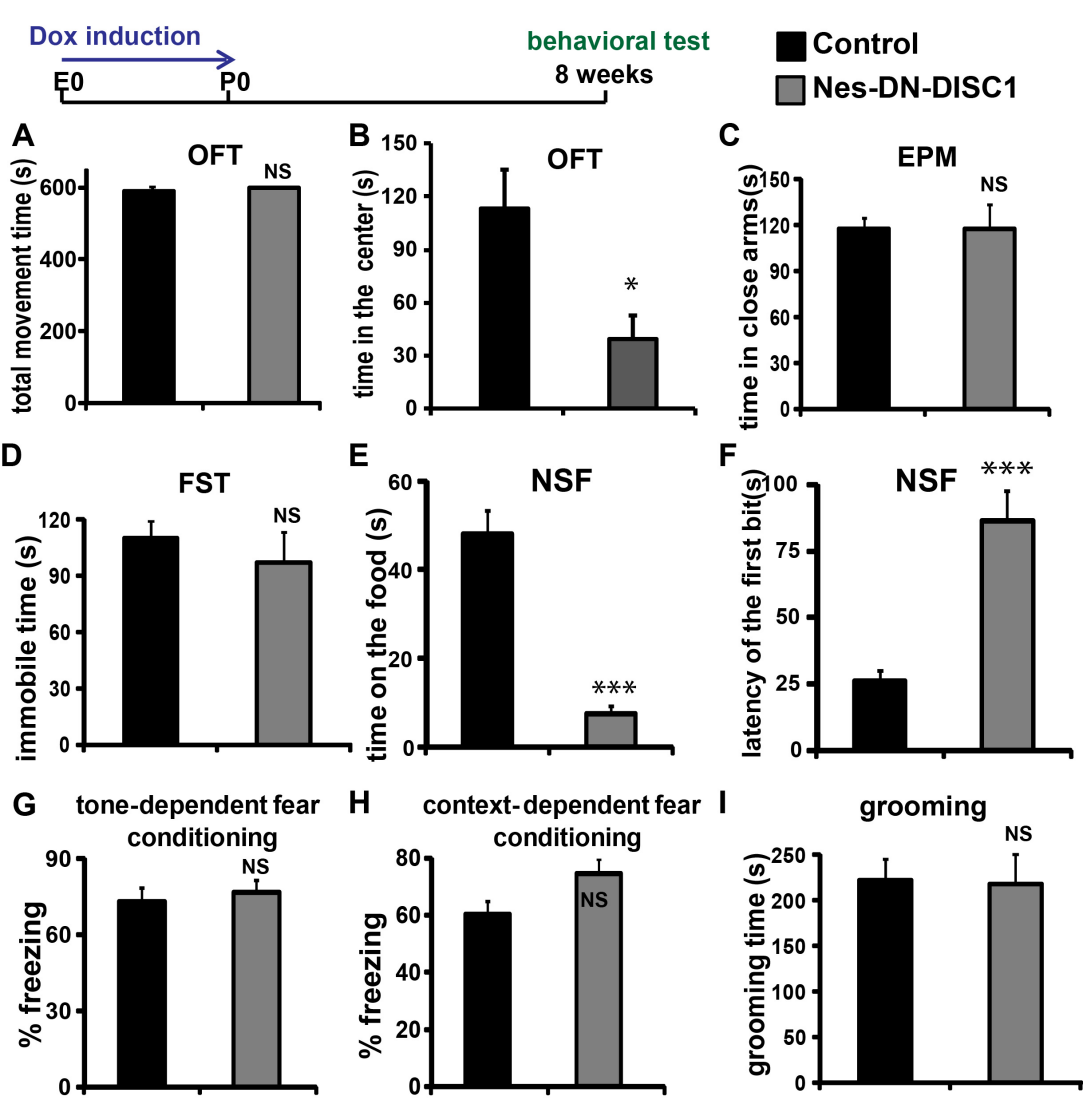
Behavioral tests on Nes-DN-DISC1 mice DN-DISC1 is induced from E0 to P0 and the mice were examined by different behavioral tests. **A.** and **B.** OFT; **C.** EPM test; **D.** FST; **E.** and **F.** NSF test; **G.** tone-dependent fear conditioning; **H.** context-dependent fear conditioning; **I.** grooming test; *n* = 7-15, *, *P <* 0.05; ***, *P <* 0.001; ANOVA test.

Our previous study showed that knockdown of *Disc1* in the dentate gyrus of adult mice leads to hyperlocomotion in the open field test (OFT), and depressive-like behavior in the forced swim test (FST) [40]. Other DISC1 models exhibit similar phenotypes [36]. Few studies have directly addressed how abnormal proliferation and differentiation of NPCs results in behavioral alterations in adulthood. We hypothesize that altered embryonic brain development, particularly in NPCs, will increase the risk for abnormal behaviors in adulthood. To test this, we induced DN-DISC1 expression from embryonic day 0 (E0) to postnatal day 0 (P0) (Figure 2), which specifically disrupted DISC1 function in embryonic NPCs. To minimize the difference between individual mice, in this study, we used the single transgenic Nes-rtTA littermates from the same pregnant mother as our control, whereas the double transgenic Nes-DN-DISC1 littermates were the mutant group, the same as the previous study [36]. Since they both were exposed to Dox with the same dose and time prenatally, this minimized the potential effect of antibiotics on behaviors.

To determine the potential influence of early DN-DISC1 exert any long-term effect on behaviors in adult mice, the littermates were off Dox food after birth (no induction) and were tested at 2 months old using a batch of behavioral tests, including the OFT, FST, elevated plus maze (EPM), grooming, fear conditioning test (FCT) and novelty suppressed feeding (NSF). The mice exhibited overall normal motor function and showed no significant differences in the total time traveled (Figure 2A) in the OFT. However, Nes-DN-DISC1 group spent much less time in the center than the control group (Figure 2B), suggesting anxiety-like behaviors. However, we didn’t detect a significant difference between two groups in the EPM test (Figure 2C, *P* = 0.97).

As DISC1 variants have been associated with MDD [51], we examined if Nes-DN-DISC1 mice show any depression-like behaviors using FST and NSF tests. Interestingly, although Nes-DN-DISC1 mice do not show depressive phenotype in response to the acute stress condition in FST (Figure 2D), they are vulnerable to chronic stress-induced depression in NSF test (Figure 2E and 2F, *P* < 0.001). Nes-DN-DISC1 mice exhibited no defect in fear memory at 8-weeks old (Figure 2G-2H) and no stereotypic grooming behaviors (Figure 2I). These results support that early genetic insults in NPCs could exhibit a long-term risk for behavioral abnormality in the adulthood even though the risk was removed.

### Effects on metabonomics after a prenatal disruption of DISC1 function in NPCs

To determine the pathological changes of Nes-DN-DISC1 mice that cause these behavioral changes in adult mice, we took advantage of an unbiased metabonomics approach, NMR, to determine the detailed metabolite changes. Pair-wise comparative orthogonal projection to latent structures with discriminant analysis (OPLS-DA) was performed using the liver and brain tissue extracts from the control and Nes-DN-DISC1 mice at different ages. Compared with control mice at postnatal one day (P1), Nes-DN-DISC1 mice exhibit lower levels of fumarate, choline and glucose in the liver (Supplementary Figure 1A). However, no significant differences of metabolites in the liver were observed between control mice and Nes-DN-DISC1 mice at P30 (data not shown). Interestingly, Nes-DN-DISC1 mice at P30 exhibited significant elevation in the levels of lipid, 3-HB, creatine, unsaturated fatty acids, and some amino acids, including glutamate, glycine, tyrosine, histidine, and phenylalanine, and hypoxanthine with a reduction in the levels of alanine, glutathione, choline, glucose, and AMP in the liver compared to age P1 (Supplementary Figure 1B), confirming that NMR can detect age-dependent changes in mouse liver metabolites.

**Figure 3:**
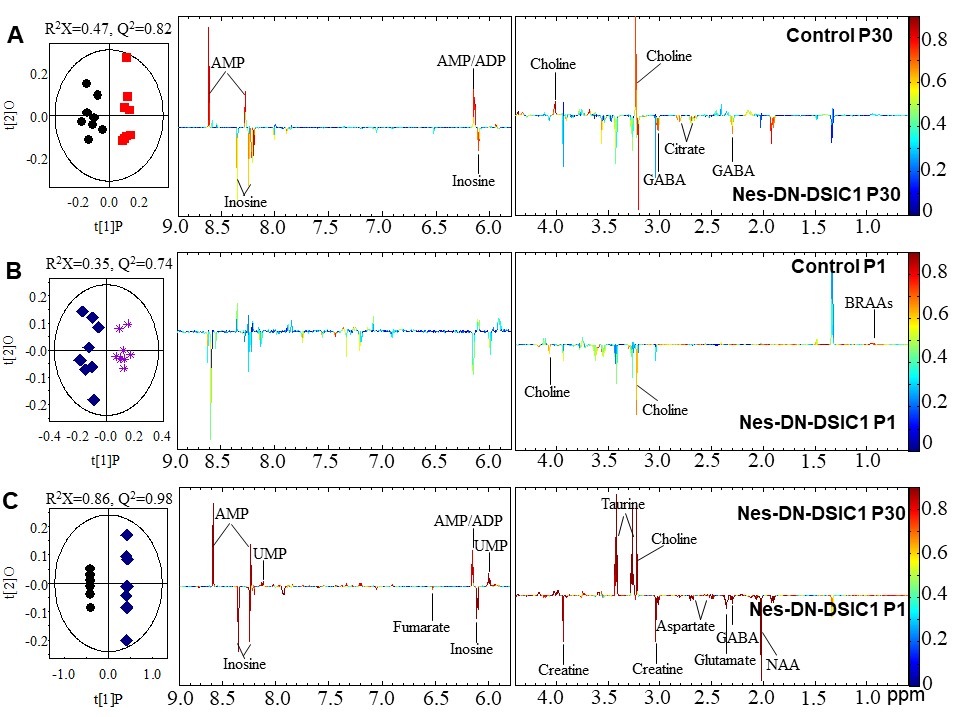
O-PLS-DA scores and coefficient-coded loadings plots for the models discriminating between the two compared groups The models are constructed from NMR spectra of aqueous brain extracts obtained at the age of P1 and P30. **A.** Comparison of control and Nes-DN-DISC1 mice at P30. **B.** Comparison of control and Nes-DN-DISC1 mice at P1. **C.** Comparison of Nes-DN-DISC1 mice between age P1 and P30. The cross-validation parameters with CV-ANOVA, Metabolite key to the numbers are shown in Supplementary Table 1.

Strikingly, in the brain, Nes-DN-DISC1 mice have higher levels of GABA, citrate and inosine but lower levels of choline and ADP/AMP than control littermates at P30 (Figure 3A). Compared with control mice at P1, Nes-DN-DISC1 mice at P1 have a lower level of choline and higher branch amino acids (BCAAs) (Figure 3B). The levels of GABA, NAA, glutamate, aspartate, creatine, fumarate and inosine are higher at P1 than those at P30 in Nes-DN-DISC1 mice while the levels of choline, taurine, AMP/UMP are lower at P1. These results suggest that DN-DISC1 expression in embryonic NPCs changes metabolites in the brain and liver and exerts a long-term effect on adult behaviors.

### Alterations of interneurons in the Nes-DN-DISC1 mice

**Figure 4:**
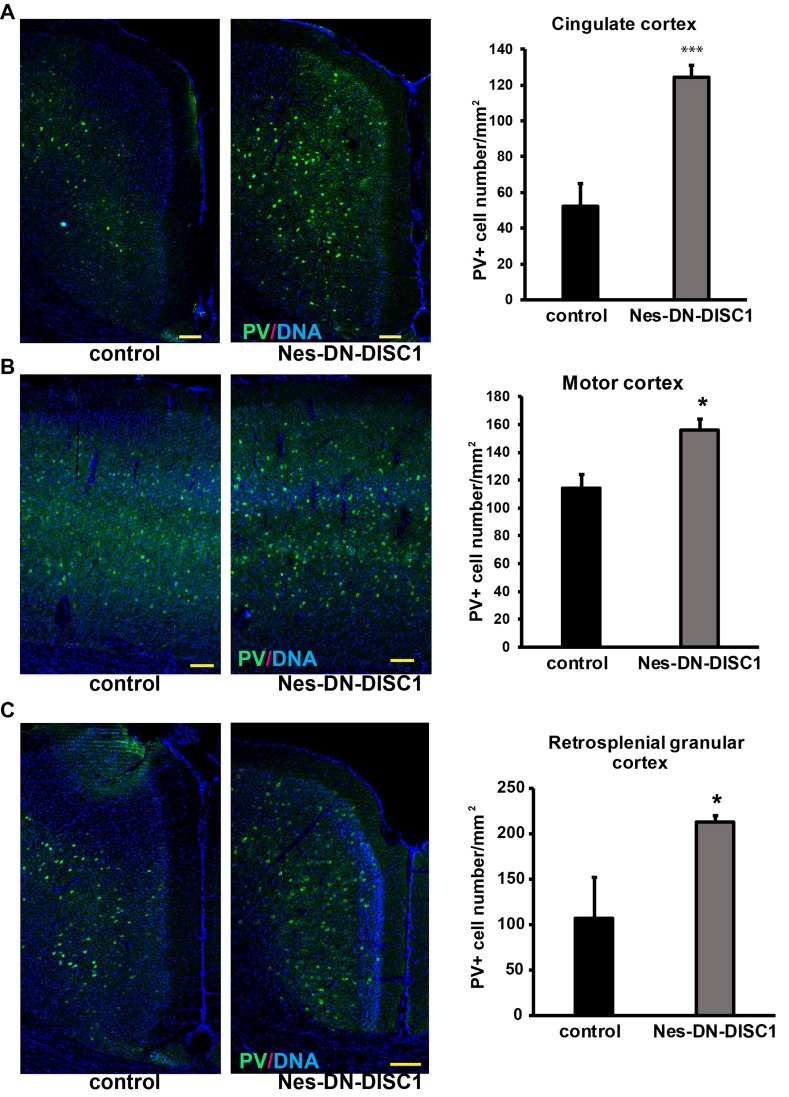
Increased PV interneuron number in different brain regions DN-DISC1 is induced from E0 to P0 and mice are sacrificed at 2 months old for PV staining (green). PV interneurons are shown as the density divided by area in following regions: **A.** the cingulate cortex, **B.** motor cortex and **C.** the retrosplenial granular cortex. *n* = 4-6. *, *p <* 0.05; ***, *p* < 0.005; *t*-test. Scale bar = 100 µm.

**Figure 5:**
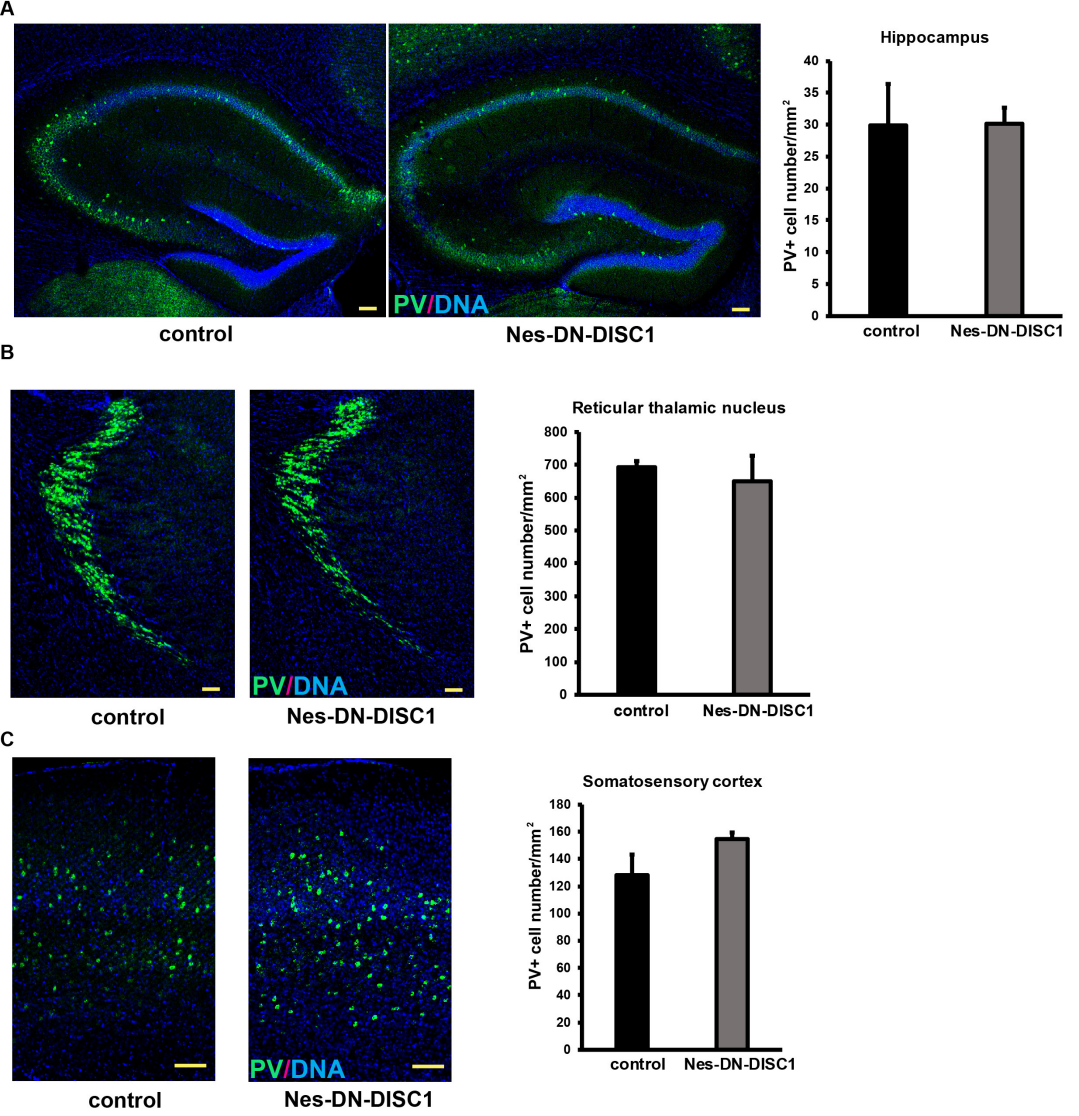
PV interneuron number is not changed in some brain regions DN-DISC1 is induced from E0 to P0 and mice are sacrificed at 2 months old for PV staining (green). PV interneurons are shown as the density divided by area in following regions: **A.** the hippocampus, **B.** the reticular thalamic nucleus and **C.** the somatosensory cortex. *n* = 4-6. Scale bar = 100 µm.

**Figure 6:**
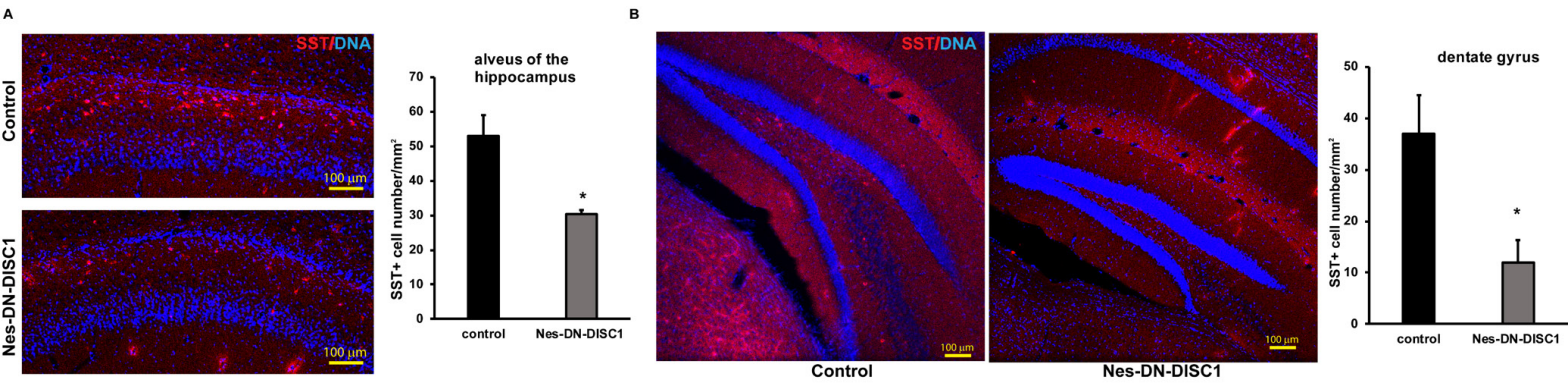
Decreased SST interneuron number in different brain regions DN-DISC1 is induced from E0 to P0 and mice are sacrificed at 2 months old for SST staining (red). SST interneurons are shown as the density divided by area in following regions: **A.** the alveus of the hippocampus, **B.** the DG. *n* = 4-6. *, *p <* 0.05; ***, *p* < 0.005; *t*-test. Scale bar = 100 µm.

**Figure 7:**
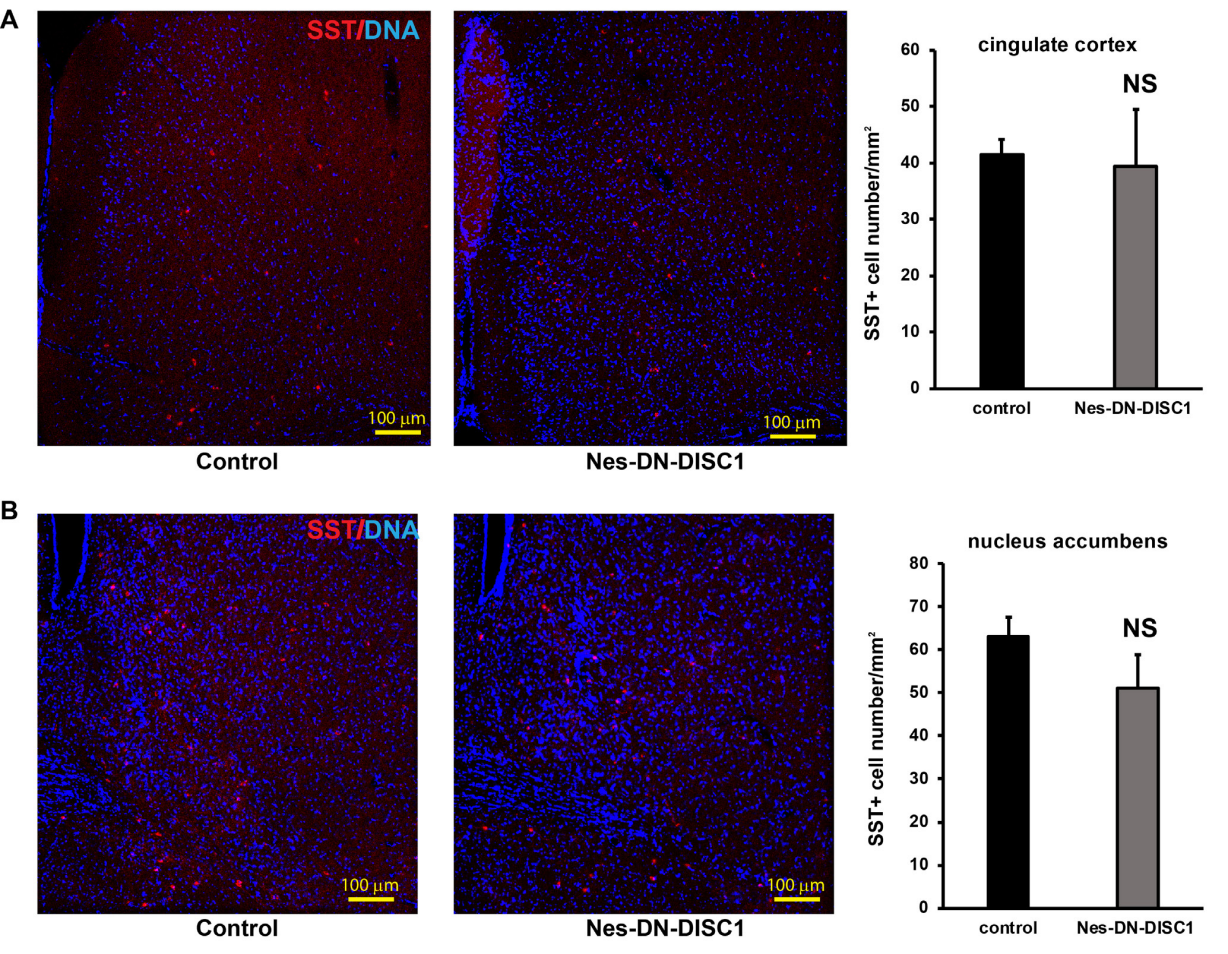
SST interneuron number is not changed in some brain regions DN-DISC1 is induced from E0 to P0 and mice are sacrificed at 2 months old for SST staining (red). SST interneurons are shown as the density divided by area in following regions: **A.** the cingulate cortex, **B.** the nucleus accumbens. *n* = 4-6. Scale bar = 100 µm.

**Figure 8:**
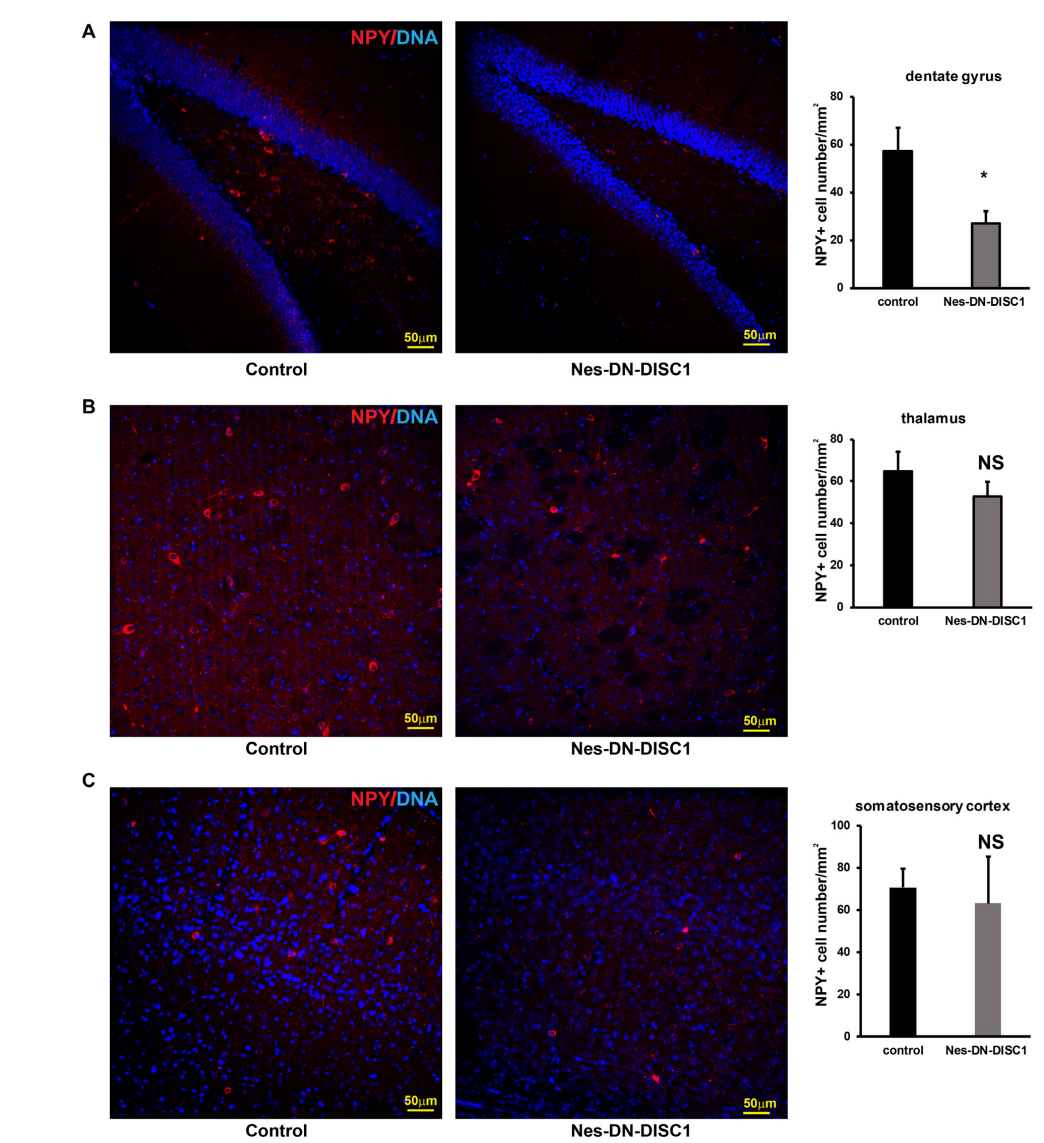
NPY interneuron number in different brain regions DN-DISC1 is induced from E0 to P0 and mice are sacrificed at 2 months old for NPY staining (red). SST interneurons are shown as the density divided by area in following regions: **A.** the DG, **B.** the thalamus, C. the somatosensory cortex. *n* = 4-6. *, *p <* 0.05; *t*-test. Scale bar = 50 µm.

To test the impact of DN-DISC1 expression in embryonic NPCs on circuitry development, we induced DN-DISC1 from E0 to P0 and then examined the gross brain structures of transgenic mice at two months old. Expression of DN-DISC1 did not cause dramatic changes in gross brain volume, lamination of cortex, and overall cell density in the cortex and hippocampus. As we detected increased GABA in the metabomics result (Figure 3), we further examined several subtypes of GABAergic interneurons different brain regions, including PV, somatostatin (SST) and neuropeptide Y (NPY) interneurons. These interneurons are important since postmortem analyses of SCZ brains show a specific defect in PV interneurons [52]. Strikingly, in contrast to other DISC1 transgenic mouse models that showed fewer PV+ interneurons [35, 37], the cell density of PV interneurons in Nes-DN-DISC1 mice was significantly increased in the cingulate cortex, retrosplenial granular cortex, and motor cortex (Figure 4A-4C), but not in the hippocampus, somatosensory cortex or reticular thalamic nucleus (Figure 5A-5C). In contrast, SST interneurons were significantly reduced in the alveus of the hippocampus and the dentate gyrus (DG) (Figure 6A-6B). However, the distribution of SST interneurons in the cingulate cortex and nucleus accumbens (Figure 7A-7B). NPY interneurons were reduced in the DG (Figure 8A), but not in the thalamus and somatosensory cortex (Figure 8B-8C). These results support that DN-DISC1 expression in embryonic NPCs alters GABAergic inhibitory neuron distribution in the adult brain.

**Figure 9:**
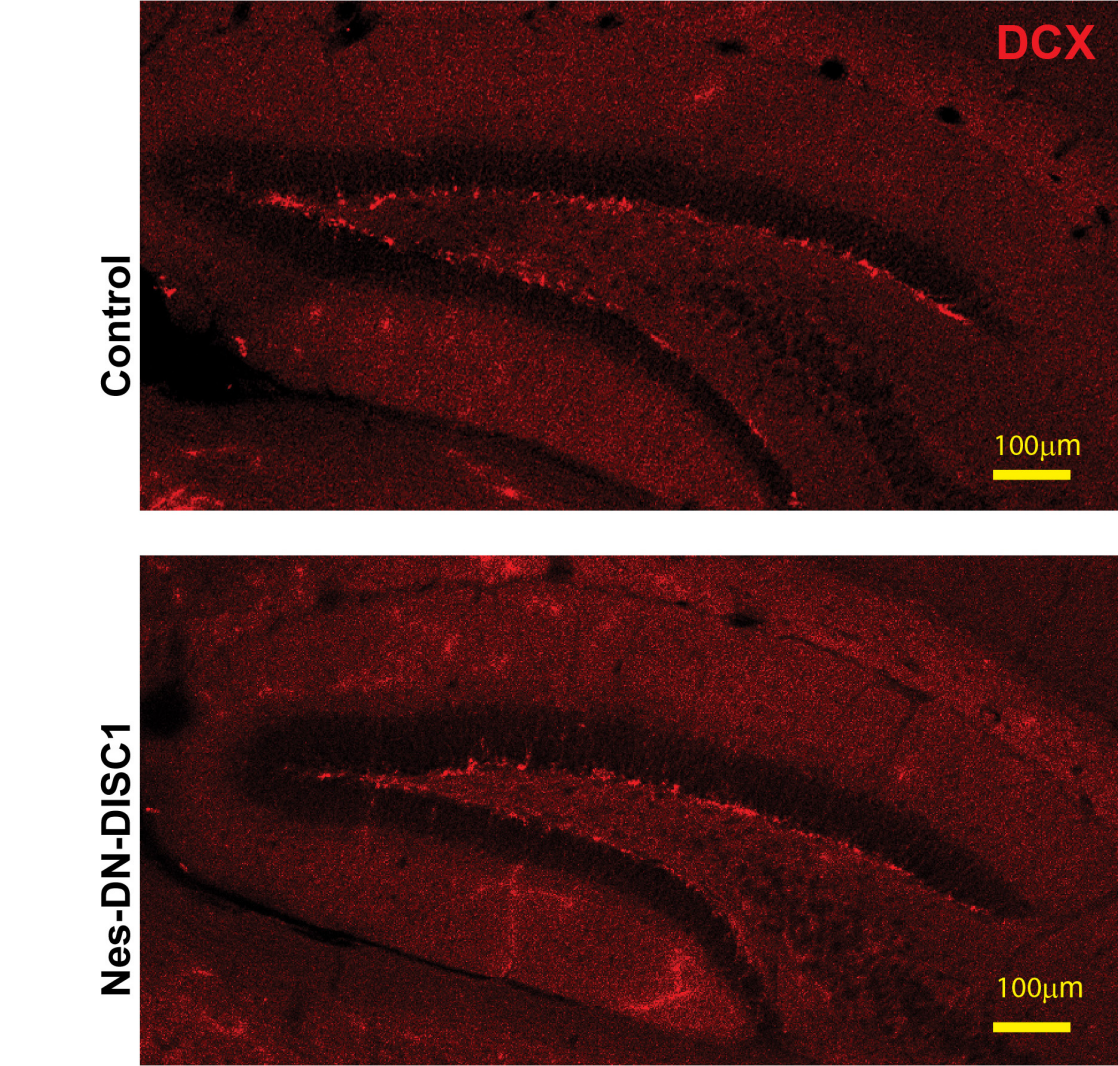
Adult neurogenesis in the DG is not changed DN-DISC1 is induced from E0 to P0 and mice are sacrificed at 2 months old for DCX staining (red). Scale bar = 100 µm.

Adult neurogenesis in the DG plays an important role in memory and depression [53-57]. We further examined the newborn neuron number using the marker, doublecortin (DCX). We found the adult neurogenesis was not changed in Nes-DN-DISC1 mice compared to control mice (Figure 9), suggesting that the depressive behavior in Nes-DN-DISC1 mice is not caused by abnormal adult neurogenesis.

### Impact on NPCs at the MGE

The results on metabonomics and interneuron distribution were surprising as other DISC1 mouse models showed decreased PV-interneurons or reduced GABA synthesis [58, 59]. In contrast, our metabonomics data suggest that early disruption of DISC1 function in NPCs leads to an enhanced GABA pathway, thereby changes animal behaviors in the adulthood. As DISC1 modulates cortical NPC function and the Wnt signaling pathway [40] that is also essential for interneuron development [60, 61], we further explored how DN-DISC1 expression affect development of interneuron progenitors.

**Figure 10:**
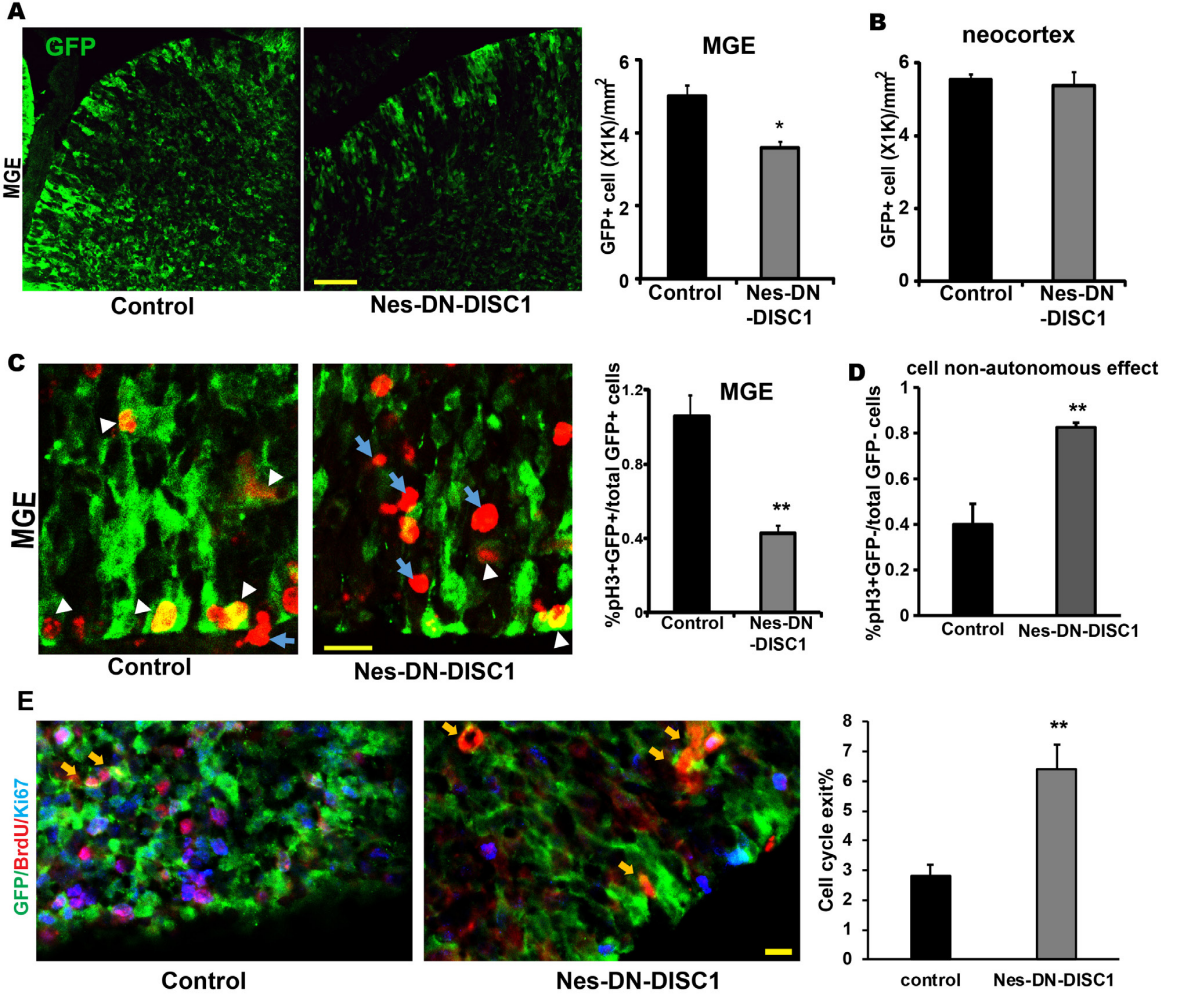
Impact of DN-DISC1 expression on NPCs at the MGE DN-DISC1 is induced by Dox at E0. Pregnant mice are sacrificed at E17. **A.** and **B.** GFP positive NPCs are significantly reduced in the MGE, but not in the neocortex. Total GFP+ cells were qualified in the MGE **A.** and in the neocortex **B.** across the whole embryonic brain at E17. The density of GFP+ cells is shown. Scale bar = 50 µm. *n* = 4-5, *, *p <* 0.05; *t*-test. **C.** DN-DISC1 reduces proliferation of NPCs in the MGE. Brain slices from E17 embryos are stained with pH3 (red), a mitotic marker, and GFP (green). GFP+ and pH3+ cells were qualified using stereological methods. White arrowheads indicate GFP+ and pH3+ cells. Blue arrows indicate pH3+ cells but GFP- cells. Scale bar = 10 µm. *n* = 4-5, **, *p <* 0.01. *t*-test. **D.** The cell non-autonomous effect of DN-DISC1 on GFP- cell proliferation is shown as the quantification of pH3+ but GFP- cells to total cells. *n* = 4-5, **, *p <* 0.01. *t*-test. **E.** BrdU was injected at E15. Mice were sacrificed at E16. The cell cycle exit index is calculated as the percentage of the GFP-positive cells that exited the cell cycle (GFP+ BrdU+ Ki67-) divided by total GFP and BrdU double positive (GFP+ BrdU+) cells. *n* = 3-4, **, *p* < 0.01, *t*-test. Scale bar = 10 µm. Yellow arrows indicate GFP+BrdU+Ki67- cells.

The cortical interneurons derived from IPCs in the GE migrate tangentially across areal boundaries of developing cortex, where they mature to form a functional network with excitatory neurons [49]. One of the consistent findings from SCZ postmortem brains is a reduction of PV interneurons [52], which is derived from medial GE (MGE). The Nes-rtTA mouse line carried the GFP reporter providing a convenient marker for labeling NPCs in both excitatory and inhibitory neural progenitors. To determine the effect of DN-DISC1 expression on interneuron progenitors, after Dox induction at E0, pregnant mice were sacrificed at E17. Interestingly, the number of GFP positive NPCs was significantly reduced in the MGE of Nes-DN-DSIC1 mice compared to control mice (Figure 10A). This decrease was not due to suppression of GFP expression by DN-DISC1 as the number of GFP+ cells in the neocortex (Figure 10B) did not change even though DN-DISC1 was also expressed in this region. We hypothesized that the decrease of GFP+ cells in Nes-DN-DISC1 mice was caused by a reduced proliferation of progenitors at the MGE. The mitotic marker, phospho-histone H3 (pH3), was used to label the nuclei of dividing cells. We quantified the GFP and pH3 double positive cells in the MGE. Consistent with our hypothesis, disruption of DISC1 function greatly reduced NPC proliferation at the MGE region ( > 60%) (Figure 10C). Intriguingly, we observed more than 2-fold increase of ectopic pH3 positive cells that were GFP negative (e. g. nestin- negative) at the MGE of Nes-DN-DISC1 mice (Figure 10D).

To test the mechanism that DN-DISC1 modulates progenitor proliferation in the MGE, we examined the cell cycle exit index. BrdU was injected at E15 into pregnant dams. Sections of E16 brains were collected and stained using anti-GFP, -BrdU, and -Ki67 antibodies (Figure 10E). GFP+/BrdU+/Ki67+ cells were in S phase at E15 and remain cycling at E16 (Figure 10E). GFP+/BrdU+/Ki67- cells (arrows) were in S phase at E15, but exited the cell cycle by E16. The cell cycle exit index represents the ratio of GFP+/BrdU+/Ki67- to total GFP+/BrdU+ cells. We observed a 2-fold increase in the cell cycle exit index in DN-DISC1 expressing embryonic brains, suggesting that the reduction of proliferating progenitors in Nes-DN-DISC1 brains probably results from increased cell cycle exit. Our results suggest that DN-DISC1 expression exerts a cell-autonomous effect to suppress proliferation of GFP+ NPCs, whereas exhibits a cell-non-autonomous effect to promote proliferation of neighboring progenitors.

**Figure 11:**
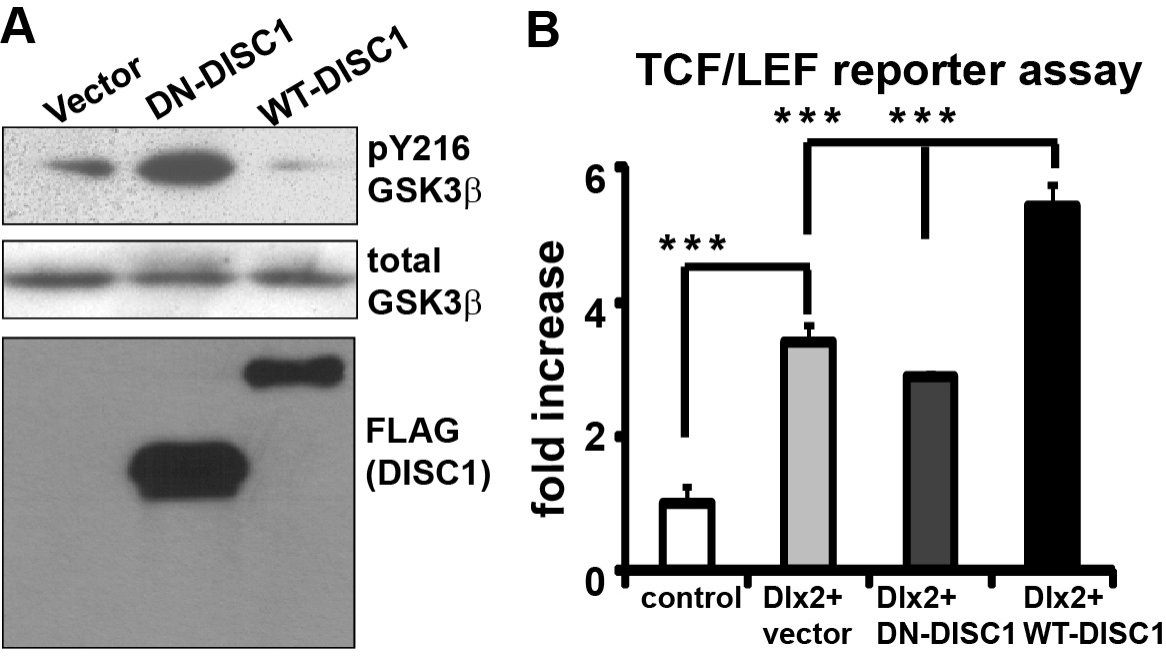
Impact of DN-DISC1 expression on the Wnt signaling **A.** Vector, WT- and DN-DISC1 were transfected into N2a cells and cell lysates were blotted with anti-pY216 GSK3β, total GSK3β and FLAG antibody. **B.** WT-DISC1 synergized with Dlx2 to activate Wnt activity. However, DN-DSIC1 impaired Dlx2-WT-DISC1 synergy in the Wnt pathway. *N* = 4, ***, *p* < 0.001, *t*-test.

Our preliminary results indicate that DN-DISC1 exerts detrimental effects on NPCs in the GE (Figure 10A). This effect is unexpected as DISC1 is highly expressed in the VZ/SVZ of all three regions of the neocortex and the GE. This suggests that IPCs in the GE express some unique factors that functionally interact with DN-DISC1 and exert this preferential impact on interneuron development. Our previous studies showed that DISC1 suppresses the active phosphorylation site Y216 on GSK3β, thereby activating Wnt signaling and promoting the proliferation in cortical NPCs. Other groups have shown that the Wnt pathway regulates interneuron differentiation [60, 62]. To examine the effect of DN-DISC1 expression on Wnt signaling, we expressed DN-DISC1 and wild type DISC1 (WT-DISC1) in N2a cells and measured pY216 levels (Figure 11A). Our data show that WT-DISC1 inhibits GSK3β activity by reducing pY216 levels, whereas DN-DISC1 significantly increases levels of pY216, suggesting that DN-DISC1 suppresses Wnt activation through its dominant negative effect on endogenous DISC1 (Figure 11A).

### Alterations of the Wnt activity by DN-DISC1

As IPCs in the GE express spatially restricted transcriptional factors, including Dlx1/2 [63, 64], Dlx5/6 [65], Nkx2.1 [66, 67], Sox6 [68], and Lhx6 [69], we *hypothesize* that DN-DISC1 interferes with these transcription factors and alters proliferation or specification of interneuron subtypes. To search for the key regulators that give rise to region-specific activation of the Wnt pathway, we uncovered a positive role of Dlx2 in the Wnt pathway. We co-expressed Dlx2 together with DN- and WT-DISC1 in N2a cells using a Wnt-dependent TCF/LEF reporter assay. Dlx2 can increases 3 folds of the reporter activity over basal levels and can synergize with WT-DISC1 in Wnt activation (Figure 11B). However, DN-DISC1 blocks this potentiating effect, suggesting that DN-DISC1 negatively regulates Dlx2-mediated Wnt activation. Thus, these results support that DN-DISC1 impedes Dlx2’s role in the interneuron development*.*

## DISCUSSION

Our study demonstrated that a prenatal genetic insult in early life could lead to long term change on brain structure, metabolism and behaviors. First, we established a novel mouse model to control spatial and temporal expression of DN-DISC1 in NPCs, which allows us to monitor the effect of risk genes on NPCs at the beginning of brain development. Second, we showed that a short-term prenatal expression of DN-DISC1 in embryonic NPCs and then off after birth was enough to cause subtle but significant behavior changes in anxiety and depression-like behaviors in adulthood. Third, using an unbiased metabonomics approach we systematically analyzed metabolic alterations in the littermates and revealed important metabolites in the brain, which lead us to identify unexpected changes of PV-interneurons in our mouse model. Forth, we examined the cellular impact of DN-DISC1 expression in IPCs at the MGE and narrow down the molecular mechanism *via* transcription factor Dlx2 in the Wnt pathway. Our study supports the “neurodevelopmental” hypothesis of mental disorders that the trajectory for dysfunctional neural circuits of psychiatric disorders is established early in life and only fully expressed in adolescence [3-5]. To the best of our knowledge, this is the first study to apply a metabonomics method in a DISC1 mouse model.

Different from the approaches used in many other DISC1 mouse models [35, 36, 42-45], we directly targeted nestin positive NPCs with inducible DN-DISC1. Moreover, using Dox inducible system, we were able to control the length of the genetic insult at the beginning of brain development selectively in NPCs (Figure 1A) but not in postnatal brains. Although this model didn’t mimic the chronic effect of the genetic mutations using the endogenous promoter, our model directly tests how prenatal genetic insults alter the development and behavioral trajectory. Interestingly, after expressing DN-DISC1 in NPCs during the prenatal period, we detected some abnormal behavioral changes in adulthood. Another way to interrupt DISC1 function in early developmental stage is to use *in utero* RNAi [70]. Niwa et al. showed that knockdown of DISC1 during in the pre and perinatal stages alters neuronal maturation and attenuates prepulse inhibition and responses to methamphetamine. The *in utero* electroporation can only affect neurons at the time of electroporation thereby has limited temporal control. Our approach will be able to influence neuronal populations generated throughout the whole embryonic period. Similar to our approach, Greenhill et al. used dox-inducible system to express c-terminal fragment of DISC1 from P7 to P9 in neurons and found a deficit of long-term potentiation (LTP) in their model [71]. Our Nes-DN-DISC1 mice show changes in anxious and depression behaviors, which are consistent with recent genetic finding of an association of DISC1 with MDD [51]. Moreover, our results together with other studies [72] suggest that the interaction of genetic risks with environmental triggers may be needed to elicit more severe psychiatric symptoms.

Our metabonomics study uncovered an unexpected result that GABA metabolite was upregulated in the mouse model. GABA is a major inhibitory neurotransmitter in the nerve system. The fine-tuning of excitatory and inhibitory inputs within cortical microcircuits is important for the proper regulation of behaviors [73]. Excitatory-inhibitory imbalance occurs in psychiatric diseases [74-78]. There have been extensive studies on inhibitory synapses, which have described the underlying mechanisms resulting in this imbalance [77, 79-84]. However, a possible cause for the imbalance may be due to a decrease in the PV-expressing GABAergic interneurons [85], which is a consistent finding based on postmortem analyses of SCZ brains. This evidence suggests that alterations in excitatory/inhibitory neuron production could result in this imbalance, yet, few studies have assessed how disruption of cortical development regarding excitatory/inhibitory neuron production leads to behavioral deregulation later in life. Our result is striking as several DISC1 animal models have shown that interneurons, particularly PV-interneurons, are decreased when DISC1 dysfunction is present chronically [35, 37]. After carefully examining the distribution of PV interneurons in our mouse model, we found that PV interneurons were selectively increased in several regions, including the cingulate cortex, retrosplenial granular cortex, and motor cortex, but not in the hippocampus, and somatosensory cortex or reticular thalamic nucleus. This is consistent with that the cingulate cortex regulates important function of emotional behaviors in depression [86]. In addition to PV interneurons, interestingly, other interneurons, such as SST and NPY neurons, were decreased in certain brain regions, suggesting a critical role of DISC1 in interneuron generation and localization. Our metabonomic extraction method is commonly used, but not all ingredients could be fully characterized after tissue processing. Even so, many metabolites were found significantly changed, which would be worthy to follow up.

While quite a lot is known about cell specification of excitatory and inhibitory neurons, the underlying mechanism for how genetic risk factors associated with psychiatric diseases affect the development of inhibitory neurons is currently elusive. DISC1 is expressed in both excitatory neuron progenitors in the VZ/SVZ of the hippocampus and neocortex [40], and inhibitory interneuron progenitors in the GE [87]. DISC1 was shown to regulate tangential migration of cortical interneurons [88]. To determine how DISC1 affects the proliferation of both excitatory neuron progenitors and inhibitory interneuron progenitors, we established a mouse line that expresses DN-DISC1 in a Dox-dependent manner within NPCs (Figure 1). This model provides a new way to test how selective DISC1 loss-of-function in NPCs generates abnormal neuronal output during development. Interestingly, we discovered that DN-DISC1 could inhibit NPC proliferation simultaneously exert cell-non-autonomous effect on neighboring cells to promote cell dividing. As nestin-expressing cells are neural stem cells with multipotential, our results suggest that DN-DISC1 limits stem cell proliferation and accelerate their differentiation into intermediate progenitors with proliferation potential by altering cell cycle progression. These ectopic dividing progenitors in the MGE could contribute to an increase of PV interneurons and a decrease of SST and NPY interneurons in specific regions.

The Wnt pathway plays a critical role to regulate interneuron development [60-62]. DISC1 is an important regulator of the Wnt pathway through regulating GSK3 [41, 89-93]. Multiple transcriptional factors, including Dlx1/2 [63, 64], Dlx5/6 [65], Nkx2.1 [66, 67], Sox6 [68], and Lhx6 [69], are crucial to determine the interneuron cell fate. We unraveled a novel role of Dlx2 in the Wnt activation and DSIC1 can potentiate Dlx2-mediated Wnt activation. However, DN-DISC1 can dampen the synergistic effect. Our data further support that DN-DISC1 could modulate the key transcription factor for interneuron development.

This study used multidisciplinary approaches involving transgenic mouse model, behavioral tests, and metabonomics methods to tackle difficult neurodevelopmental issues that cannot be addressed by human studies. Thus, our research provides a different strategy to model the pathophysiology of mental illness, which will deepen our understanding of the developmental origins of mental diseases.

## MATERIALS AND METHODS

### Animals

Tg(Nes-rtTA-ires-GFP)PN9Kern mouse (Nes-rtTA-GFP) [48] was kindly provided by Dr. Kernie (U. T. Southwestern Medical Center). Tg(tetO/CMV-DISC1*)70 Plet mouse (tetO-DN-DISC1) was kindly provided by Dr. Mikhail Pletnikov (Johns Hopkins University School of Medicine) [36]. All mice were housed at controlled room temperature (22-24°C) with a 12-hour light (light on 7:00 am to 6:00 pm) and 12-hour dark cycle. Mice had *ad libitum* access to food and water. The animal experiments were approved by the IACUC Committee of the Pennsylvania State University.

Nes-rtTA-GFP mice were mated with tetO-DN-DISC1 mice to generate Nestin-rtTA-GFP; tetO-DN-DISC1 (Nes-DN-DISC1) double transgenic line. The littermates were genotyping using rtta pimers (5’- GGA CAA GAG CAA AGT CAT AAA CGG-3’ and 5’- TTC GTA CTG TTT CTC TGT TGG GC-3’) for Nes-rtTA-GFP mice and TRE-DISC1 primers (TRE-CMV-F4: 5’-gacctccataga agacaccgggac-3’, and TRE-hDISC1-R2: 5’-tgagctgaatcccaaagtgcgccg-3’) for TetO-DN-DISC1 mice.

### DNA constructs

Full length and DN-human DISC1 was amplified by PCR and subcloned into the 3XFLAG expression vectors [40]. Super 8XTOPFLASH (which contains 8 copies of the TCF/LEF binding site), a gift from Dr. R. Moon (University of Washington, WA) and a Renilla-Luc-TK reporter (pRL-TK, Promega) were used for testing TCF transcriptional activity.

### Immunohistochemistry and immunobloting

Adult mice were anesthetized with Avertin (200 mg per kg of body weight) and perfused intracardially with 150ml saline, then followed by 150ml of 4% paraformaldehyde in phosphate buffer (PBS). Brains were removed, post-fixed in 4% paraformaldehyde at 4°C for overnight. Fifty µm coronal sections were cut using a vibrotome for adult brains. Embryonic brains at E17 were drop-fixed in 4% paraformaldehyde/PBS at 4°C for overnight, dehydrated in 30% sucrose and sliced in the cryostat instrument (Leica) at the thickness of 10 µm. Brain sections were blocked with 5% normal donkey serum in PBS with 0.3% Triton X-100 for 60 min. Brain sections were incubated with primary antibodies, chicken anti-GFP (1:1000, Aveslabs), rabbit anti-PV (1:500, Santa Cruz), rabbit anti-NPY (1:500, Santa Cruz), rat anti-SST (1:500, Millipore), rabbit anti-Ki67 (1:500, GeneTex), goat anti-Sox2 (1:500, Santa Cruz), rabbit anti-phosph-Histon H3 ser10 (pH3), or mouse anti-nestin (1:50, DSHB) antibodies in fresh blocking solution and incubate with brain slides for overnight at room temperature. After washing with PBS, brain slides were incubated with secondary antibodies conjugated with fluorescent groups (Thermo-Fisher). Brain slides were mounted to glass slides and photographed by Zeiss Pascal confocal microscope (Carl Zeiss, USA). Zeiss LSM image browser software (Carl Zeiss, USA) and Image J was used for analysis of images. The number of pH3 positive cells was counted from sections and presented as the percentage of the GFP-labeled cells. PV positive cells were counted in sections and presented as density divided to the region area. Stereology analysis was used to examine the cell distribution. Basically, brain sections were isolated from one in every 6 sections across the whole brain and were stained with different antibodies. Over 200 positive cells per brain (*n* = 3-6 brains) were counted.

Brain lysates from induced or non-induced brains at E17 were lysed and blotted with mouse anti-myc (DSHB) and mouse anti-actin (GenScript) antibodies. N2a cells were transfected with vector, FLAG-tagged WT-DISC1 and DN-DISC1 for 48 hours and protein concentration was determined with the assay kit (Bio-Rad Laboratories). Western blot was performed as described previously [94] using rabbit anti-pY216 GSK3β, rabbit anti-total GSK3β (Cell Signaling) and mouse anti-FLAG epitope (Sigma).

### Luciferase assay

N2a cells were seeded into 24-well plates and transfected with 0.2 µg of 8XTOPFLASH reporter and 0.05 µg of pRL-TK, *0.4* µg WT-DISC1 or DN-DISC1 and 0.4 µg mouse DLX2 [95] using *polyethylenimine*. 24 hours after transfection, TCF reporter activity was measured using the Dual-Luciferase Assay System (Promega).

### Behavioral tests

The pregnant female mice were fed with Dox containing food (Bio-Serv, 200 mg/kg) from the beginning of pregnancy on embryonic day 0(E0). The Dox food was removed right after the littermates were delivered on postnatal day 0 (P0). The single transgenic *Nes-rtTA* littermate mice from the same pregnant mother were used as control. The control and Nes-DN-DISC1 littermates (6-15 mice each group) were tested in different behavioral tests at age of P60. The mice were kept in their cages and acclimated to the behavior testing room 1 h before each test. We recorded each trial with an EthoVision XT video tracking system and software (Noldus).

### Open field test

Mice were habituated to the testing room for 30 min before the experiment. Each mouse was placed in the center of an arena (black floor: 50x50 cm divided into 25 of 10x10 cm squares, walls: 50cm high). Mice were allowed to move freely during a 5 min trial, and the mice were videotaped. The center region was defined as the 30x30 cm area. Percent time spent in the center and the periphery of the open field test arena was quantified as an index of anxiety. For overall locomotor activity, total traveled time was calculated.

### Forced swimming test

The forced swimming test was performed as previously described [40]. After habituation time, mice were placed individually in transparent glass beaker (15 cm high, 8 cm diameter) containing 800ml water at 25°C. Mice were videotaped for 5 min, and the immobility time (which refers to the time of the passive floating of mice) were recorded.

### Elevated plus maze

The elevated plus maze test was used to assess the exploratory and anxiety-like behaviors [96]. The elevated plus maze used was (+) shaped, 50 cm elevated from the floor with two open arms and two enclosed arms (30 cm length, wide 5cm). Mice were placed in the center of the maze and, allowed to freely move into the four arms of the maze for 5 minutes. The mice were videotaped and the times spent in open and closed arms were scored.

### Novelty suppressed feeding

Novelty Suppressed Feeding is a test for chronic depression. Mice were weighed and food deprived from their cage 18 hours before the test. Each mouse was placed in the corner of a chamber that is 18” by 24” covered in bedding. At the center of the chamber was a small amount of food placed on a white Whatman filter paper that is 5 cm in diameter. The center area was brightly lit. The animals were allowed to move around freely for the next 6 minutes, and the time the animal took to start eating the food were measured, as well as the total time spent eating.

### Fear conditioning test

In the training session, each mouse was adapted in the fear conditioning instrument for 5 min. After the habituation, the mouse in the testing chamber will receive a 2-sec, 0.55-mA foot shock every 80-sec [97]. The mouse was removed immediately after the third shock. During the contextual testing, the freezing behavior of each mouse was recorded in the testing chamber for two groups.

### Grooming test

Grooming test was used to measure the repetitive behaviors [98]. The face of each mouse was misted with a spray of distilled water and mouse was placed into an empty cage with no bedding. The animals were videotaped for 15 min. Total grooming time was measured manually from the video. The first 5 min was excepted from the analysis.

### Statistical analysis

Data were analyzed using Excel are expressed as means ± standard error of the mean (SEM). Significances between the experimental group and control group were analyzed by Student’s *t-test* and ANOVA.

### Sample preparation for NMR spectroscopy

Brain and liver were collected immediately following CO_2_ asphyxiation on day 1 or 30. All samples were stored at -80°C until analysis. Brain or liver tissues (∼50 mg) were extracted three times with 600 μl of a precooled methanol−water mixture (2/1, v/v) using the Precellys tissue homogenizer (Bertin Technologies, Rockville, MD). After centrifugation at 11180g for 10 min at 4 °C, the combined supernatants were dried. Each of the aqueous extracts was separately reconstituted into 600 μl of phosphate buffer (K_2_HPO_4_/NaH_2_PO_4_, 0.1 M, pH 7.4, 50% v/v D_2_O) containing 0.005% sodium 3-trimethylsilyl [2,2,3,3-d^4^] propionate (TSP-d4) as chemical shift reference. Following centrifugation, 550 μl of each extract was transferred into a 5 mm NMR tube for NMR analysis [99, 100].

### ^1^H NMR spectroscopy

^1^H NMR spectra of aqueous extracts were acquired at 298 K on a Bruker Avance III 600 MHz spectrometer (operating at 600.08 MHz for 1H and at 150.93 MHz for 13C) equipped with a Bruker inverse cryogenic probe (Bruker Biospin, Germany). A typical one-dimensional NMR spectrum was acquired for each of all samples employing the first increment of the NOESY pulse sequence (NOESYPR1D). To suppress the water signal, a weak continuous wave irradiation in the NOESY method was applied to the water peak during the recycle delay (2 s). The 90° pulse length was adjusted to approximately 10 μs for each sample, and 64 transients were collected into 32 k data points for each spectrum with a spectral width of 20 ppm. To facilitate NMR signal assignments, a range of 2D NMR spectra was acquired and processed for selected samples, including 1H−1H correlation spectroscopy (COSY), 1H−1H total correlation spectroscopy (TOCSY), 1H−13C heteronuclear single quantum correlation (HSQC), and 1H−13C heteronuclear multiple bond correlation spectra (HMBC). Mixing time was 100 ms in the 1D NOESY and 2D TOCSY experiments.

### Spectral data processing and multivariate data analysis

All free induction decays (FID) were multiplied by an exponential function with a 1 Hz line broadening factor prior to Fourier transformation. The spectra were referenced to TSP-d4 at δ 0.00 when TSP-d4 was present in the liver or brain extracts. ^1^H NMR spectra were corrected manually for the phase and baseline distortions, and the spectral region δ 0.50−9.50 was integrated into regions with equal width of 0.004 ppm (2.4 Hz) using the AMIX software package (V3.8, Bruker-Biospin). Region δ 4.60−5.15 was discarded by imperfect water saturation. Each bucketed region was then normalized to the total sum of the spectral integrals to compensate for the overall concentration differences prior to statistical data analysis. Multivariate data analysis was carried out with SIMCA-P+ software (version 13.0, Umetrics, Sweden) as described [101, 102]. Briefly, principal component analysis (PCA) and orthogonal projection to latent structures with discriminant analysis (OPLS-DA) were conducted on the NMR data. The OPLSDA models were validated using a 7-fold cross validation method, and the quality of the model was described by the parameters R^2^X and Q^2^ values (Figure 3). After back-transformation of the loadings generated from the OPLSDA, color-coded correlation coefficient loading plots (MATLAB, The Mathworks Inc.; Natick, MA) were employed to indicate the significance of the metabolite contribution to the class separation with a “hot” color (e.g., red) being more significant than a “cold” color (e.g., blue). In this study, a cutoff value of |r| > 0.653 (r > +0.653 and r < −0.653) was chosen for the correlation coefficient as significant based on the discrimination significance (*P* ≤ 0.05). Metabolite key to the numbers are shown in Supplementary Table 1. Data were collected from 8 mice each genotype.

## SUPPLEMENTARY MATERIALS FIGURE AND TABLE




