# Supplementary material for: A prenatal interruption of DISC1 function in the brain exhibits a lasting impact on adult behaviors, brain metabolism, and interneuron development
Source: Oncotarget. 2017 Sep 28;8(49):84798–817. doi: 10.18632/oncotarget.21381 (PMC5689574; doi:10.18632/oncotarget.21381)
Supplement: Supplementary file 1 [file oncotarget-08-84798-s001.pdf]

A prenatal interruption of DISC1 function in the brain exhibits a lasting impact on adult behaviors, brain metabolism, and interneuron development

SUPPLEMENTARY MATERIALS

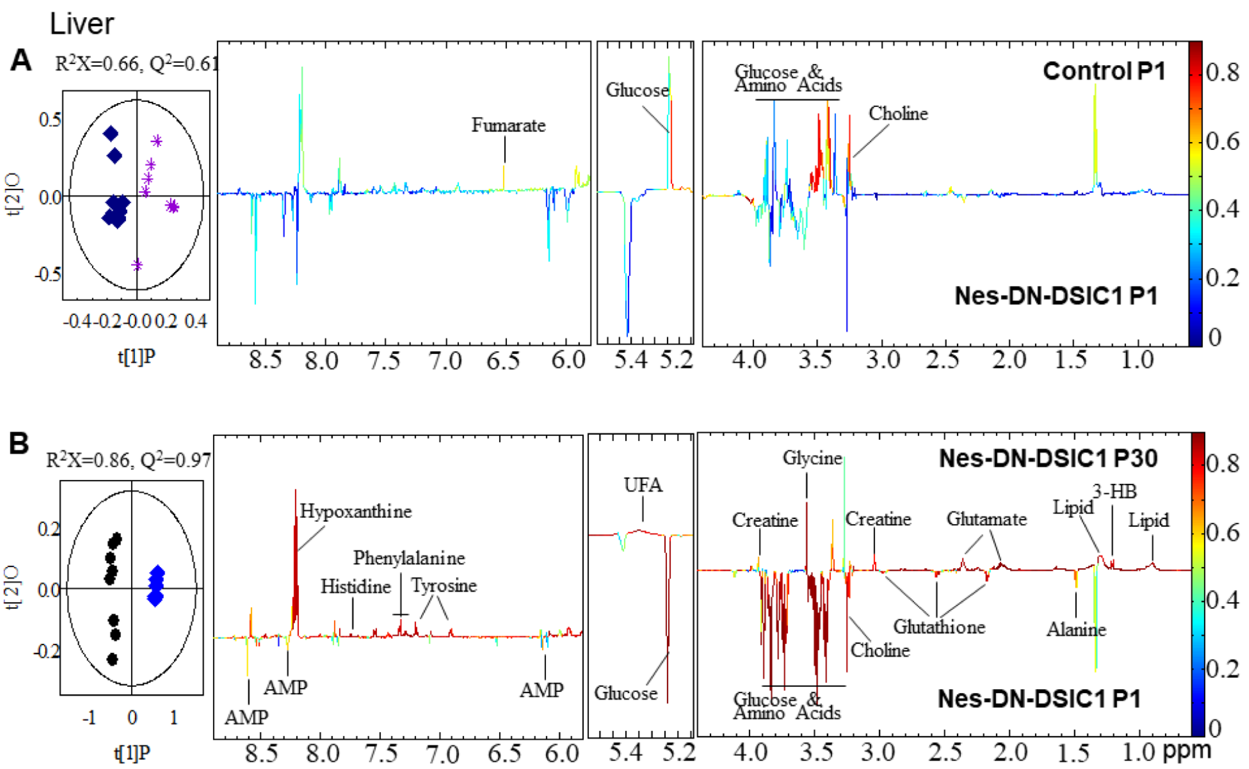

Figure S1. O-PLS-DA scores and coefficient-coded loadings plots for the models discriminating between the control group (black squares) and Nes-DN-DISC1 mice (red dots). The models are constructed from NMR spectra of aqueous liver extracts obtained at age of P1 and P30. A. Comparison of control and Nes-DN-DISC1 mice at P1. B. Comparison of Nes-DN-DISC1 mice between age P1 and P30. The cross-validation parameters with CV-ANOVA, Metabolite key to the numbers are shown in Table S1.

For Supplementary Table see in Supplementary Files
