# Supplementary material for: A prenatal interruption of DISC1 function in the brain exhibits a lasting impact on adult behaviors, brain metabolism, and interneuron development
Source: Oncotarget. 2017 Sep 28;8(49):84798–817. doi: 10.18632/oncotarget.21381 (PMC5689574; doi:10.18632/oncotarget.21381)
Supplement: Supplementary file 2 [file oncotarget-08-84798-s002.pdf]

Table S1. ^{1}H NMR chemical shifts for metabolites assigned in liver and brain tissue extracts.

key	metabolites	moieties	δ ^{1}H (ppm) and multiplicity[a]	Samples[b]
1	Lipid	CH_3, $(CH_2)_n$, CH_2-C=C, CH_2-C=O,C-CH_2-C=,-CH=CH-	0.89(m), 1.27(m), 2.0(m), 2.3(m), 2.78(m), 5.3(m)	L, B
2	Isoleucine	αCH, βCH, γCH_3, δCH_3	3.65(d), 1.95(m), 0.99(t), 1.02(d)	L, B
3	Leucine	αCH, βCH_2, γCH_3, δCH_3	0.94(d), 3.72(t), 1.96(m), 0.91(d)	L, B
4	Valine	αCH, βCH, γCH_3	3.6(d), 2.26(m), 0.98(d), 1.04(d)	L, B
5	D-3-hydroxybutyrate	CH, CH_2, γCH_3, CH_2	4.16(dt),2.41(dd),1.20(d),2.31(dd)	L
6	Lactate	αCH, βCH_3	4.11(q), 1.32(d)	L, B
7	Alanine	αCH, βCH_3	3.77(q), 1.48(d)	L, B
8	Acetate	CH3	1.91(s)	L, B
9	Lysine	αCH, βCH, γCH3	3.74(t), 1.90(m), 1.72(m)	L, B
10	Glutamate	αCH, βCH2, γCH2	2.08(m), 2.34(m), 3.75(m)	L, B
11	Glutamine	αCH, βCH2, γCH2	2.15(m), 2.44(m), 3.77(m)	L, B
12	Glutathione	CH_2, CH_2, S-CH_2, N-CH, CH	2.16(m), 2.55(m), 2.95(dd), 3.78(m), 4.56(q)	L
13	Succinate	CH_3	2.41(s)	L, B
14	Pyruvate	CH_3	2.38(s)	L, B
15	Aspartate	αCH, βCH2, γCH2	3.90(m), 2.68(m), 2.82(dd)	L, B
16	Choline	$N(CH_3)_3$, OCH_2, NCH_2	3.2(s), 4.05(t), 3.51(t)	L, B
17	Phosphocholine(PC)	$N(CH_3)_3$, OCH_2, NCH_2	3.22(s), 4.21(t), 3.61(t)	L, B
18	Glycerophosphocholine	$N(CH_3)_3$, OCH_2, NCH_2	3.22(s), 4.32(t), 3.68(t)	L, B
19	TMAO	CH_3	3.27(s)	L
20	Taurine	S-CH_2, N-CH_2	3.26(t), 3.40(t)	L, B
21	Glucose & amino acids	αCH resonances	3.3-3.9	L
22	Triglycerides	CH	4.08(m), 4.21(m), 5.18(m)	L
23	α-Glucose	1-CH	5.23(d)	L
24	Glycogen	1-CH	5.38-5.45(m)	L
25	Unsaturated fatty acid	CH=CH	5.3(m)	L
26	Uridine	11-CH, 7-CH, 12-CH,	7.88(d), 5.92(d), 5.9(d), 4.36(m),	L, B

		6-CH, 5-CH, 4-CH, CH_2, CH_2	4.24(t)	
27	Uridine diphosphate (UDP)	C6,ring, C1'H,ribose<br>C5,ring, C3'H,ribose<br>C5'H,ribose, 4'H,ribose	7.94(d)<br>5.98(d)<br>5.97(d)	L, B
28	Inosine	14-CH, 1-CH, 8-CH, 4'-CH, 5'-CH, CH_2(1/2), CH_2(1/2)	8.34(s), 6.09(d), 8.24(s), 4.76(t), 4.47(m)	L, B
29	Adenosine monophosphate (AMP)	2-H, 8-H, 2'-H	8.61(s), 8.27(s), 6.15(d)	L, B
30	Fumarate	CH	6.53(s)	L, B
31	Tyrosine	CH, CH	6.89(dd), 7.18(dd)	L, B
32	Histidine	2-CH, 4-CH, CH_2	7.75(t), 7.08(d), 6.05(d)	L, B
33	Phenylalanine	Ring-CH	7.40(m), 7.33(m), 7.35(m)	L, B
34	Uracil	1-CH, 2-CH	5.81(d), 7.54(d)	L, B
35	Xanthine	CH	7.88(s)	L, B
36	Uridine monophosphate (UMP)	C6,ring, C1'H,ribose<br>C5,ring, C3'H,ribose<br>C5'H,ribose, 4'H,ribose	8.12(d)<br>5.98(d)<br>5.97(d)	L
37	Hypoxanthine	CH, CH	8.20(s), 8.21(s)	L, B
38	Nicotinamide	2-CH, 6-CH, 4-CH, 5-CH	8.93(s),8.62(d), 8.25(d),7.60(dd),	L, B
39	Betaine	CH_2, CH_3	3.27(s), 3.93(s)	L
40	Bile acid	CH_3	0.73(m)	L
41	Inosine-5'-monophosphate (5'-IMP)	2-H, 8-H, 2'-H	8.54(s), 8.28(s), 6.15(d)	L, B
42	Formate	CH	8.45(s)	L, B
43	Adenosine	14-CH	8.34(s)	L, B
44	Creatine	CH, CH	3.03(s), 3.93(s)	L, B
45	Glycine	CH	3.57(s)	L, B
46	N-acetyl-aspartate (NAA)	CH	2.02(s)	B
47	U2	CH	2.30(t)	B
48	U3		3.63(t)	B
49	Guanosine	CH	8.0(s)	B

50	γ-aminobutyric acid (GABA)	CH2<br>CH	2.3(m),<br>3.0(m)	B

[a] Key: s, singlet; d, doublet; t, triplet; q, quartet; m, multiplet; dd, doublet of doublet.

[b] Liver (L) and Brain (B) aqueous extracts.

U: unknown
